# Modified posteromedial approach for treatment of posterior pilon variant fracture

**DOI:** 10.1186/s12891-016-1182-9

**Published:** 2016-08-05

**Authors:** Yukai Wang, Jianwei Wang, Cong feng Luo

**Affiliations:** Trauma Service III, Shanghai Sixth People’s Hospital, affiliated with Shanghai Jiaotong University, N. 600 Yishan R. d, Xuhui Distict Shanghai, China

**Keywords:** Ankle fractures, Posterior pilon, Posterior malleolus, Posteromedial approach, Posterolateral approach, Tibial plafond

## Abstract

**Background:**

Posterior pilon variant fracture is a recently described posterior malleolus fracture characterized by the involvement of both posterolateral and posteromedial malleolar fragment. The associated surgical approach remains controversial. The aim of this study was to present the application of modified posteromedial approach in the treatment for posterior pilon variant fracture.

**Methods:**

Sixteen patients were identified with posterior pilon variant fractures. All fractures were operated via modified posteromedial approach. Fragment length ratio, area ratio and height were measured as morphologic assessments. The clinical outcome was evaluated with American Orthopaedic Foot & Ankle Society ankle-hind foot score and visual analogue scale. Radiological images were evaluated using osteoarthritis-score.

**Results:**

According to the radiological measurements, the average fragment length ratio of posteromedial and posterolateral fragment was 25.3 and 31.5 % respectively. All fractures healed within a mean period of 13.1 weeks without malalignment or articular step-off. Fourteen patients were followed up, and all achieved good or excellent ankle function. The average score of American Orthopaedic Foot & Ankle Society and visual analogue scale at rest, motion and weight bearing walking was 85.6 and 0.25, 0.81, 1.31 respectively.

**Conclusion:**

Modified posteromedial approach provides an alternative surgical treatment for posterior pilon variant fractures, and the short-term outcome was good.

**Electronic supplementary material:**

The online version of this article (doi:10.1186/s12891-016-1182-9) contains supplementary material, which is available to authorized users.

## Background

Posterior pilon, which has drawn attention over recent years, is considered as a variant of posterior malleolar fracture [1–15]. The term was first given by Hansen et al. [1] in 2000, and later reported by Weber [2], which is described as posterior malleolar fractures extending into posterior colliculus, indicating the presence of posteromedial (PM) fragment. Different from standard trimalleolar and Volkmann fracture, posterior malleolar fracture in ‘posterior pilon variant’ split into PM and PL fragment [3, 4, 8]. To date, taking both fracture morphology and injury mechanism into consideration, “posterior pilon variant” as we adopted in this article, may indicate an independent fracture pattern, which requires special attention in surgical approach and appropriate fixation.

Despite the rising interests, question remains what is the optimal solution to posterior pilon variant, as current evidence relevant with treatment is limited. We consider it necessary that anatomical reduction should be achieved regardless of the size of posterior tibial plafond fragment(s), as talar subluxation may persist without surgical management of PM fragment [2, 12]. Moreover, no consensus has been reached on the best way to approach posterior pilon variant, though posterolateral approach has been widely accepted in direct reduction and fixation of posterior malleolus [5, 6, 11, 15, 16].

The purpose of this study was to report on the use of a modified posteromedial approach in surgical treatment for posterior pilon variant fracture, specifically the ability to expose and stabilize the posteromedial and posterolateral fragments. The outcomes associated with the technique and the morphologic characteristics of posterior pilon variant are reported as well.

## Methods

**Fig. 1:**
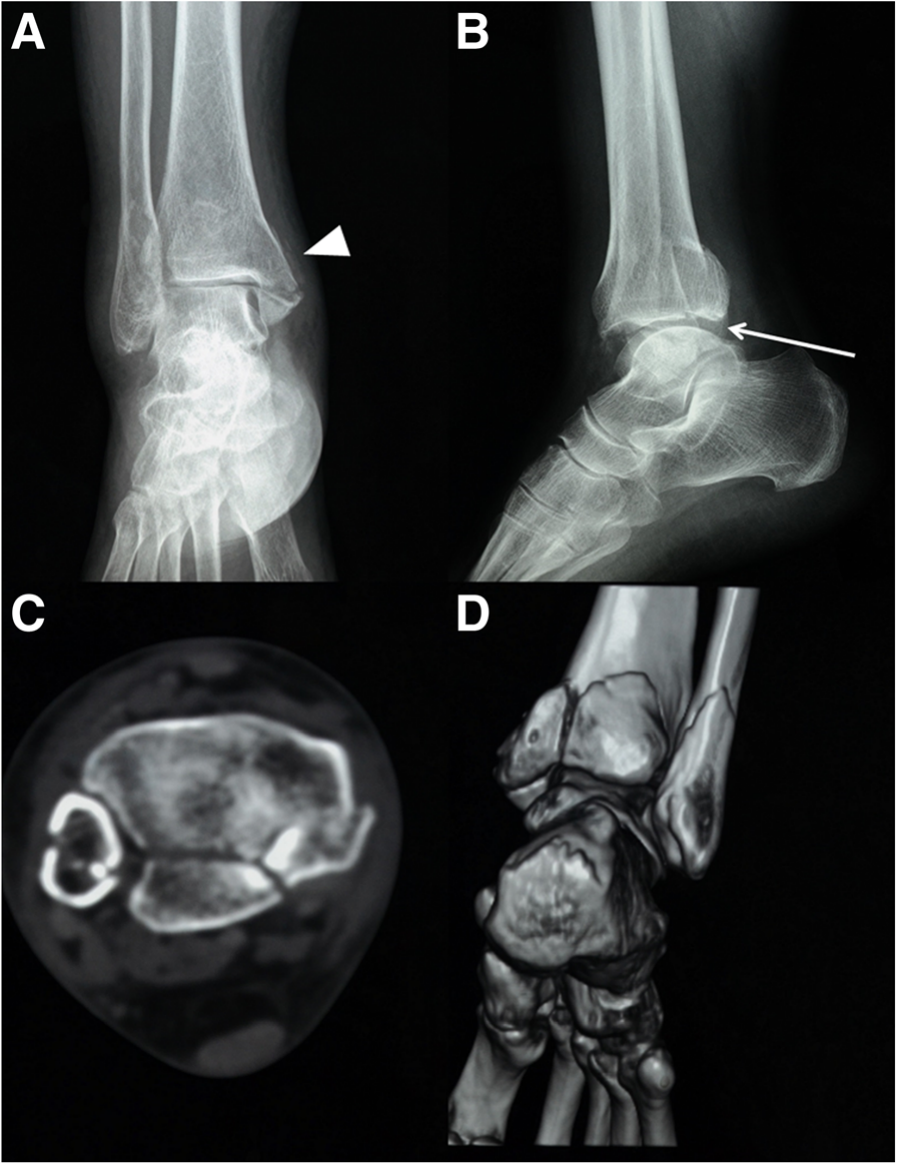
Preoperative radiographs of case 3. **a**-**b** The *Arrow Head* showing the “double contour” sign on AP view indicates the existence of PM fragment. The *Arrow* showing the “double joint line” sign on lateral view indicates the proximally displaced posterior tibial plafond. In together with the two signs, posterior pilon fracture is in highly suspicion. **c**-**d** CT scan and reconstruction of the same patient provides a better view of the location and size of the PM, PL fragment

**Fig. 2:**
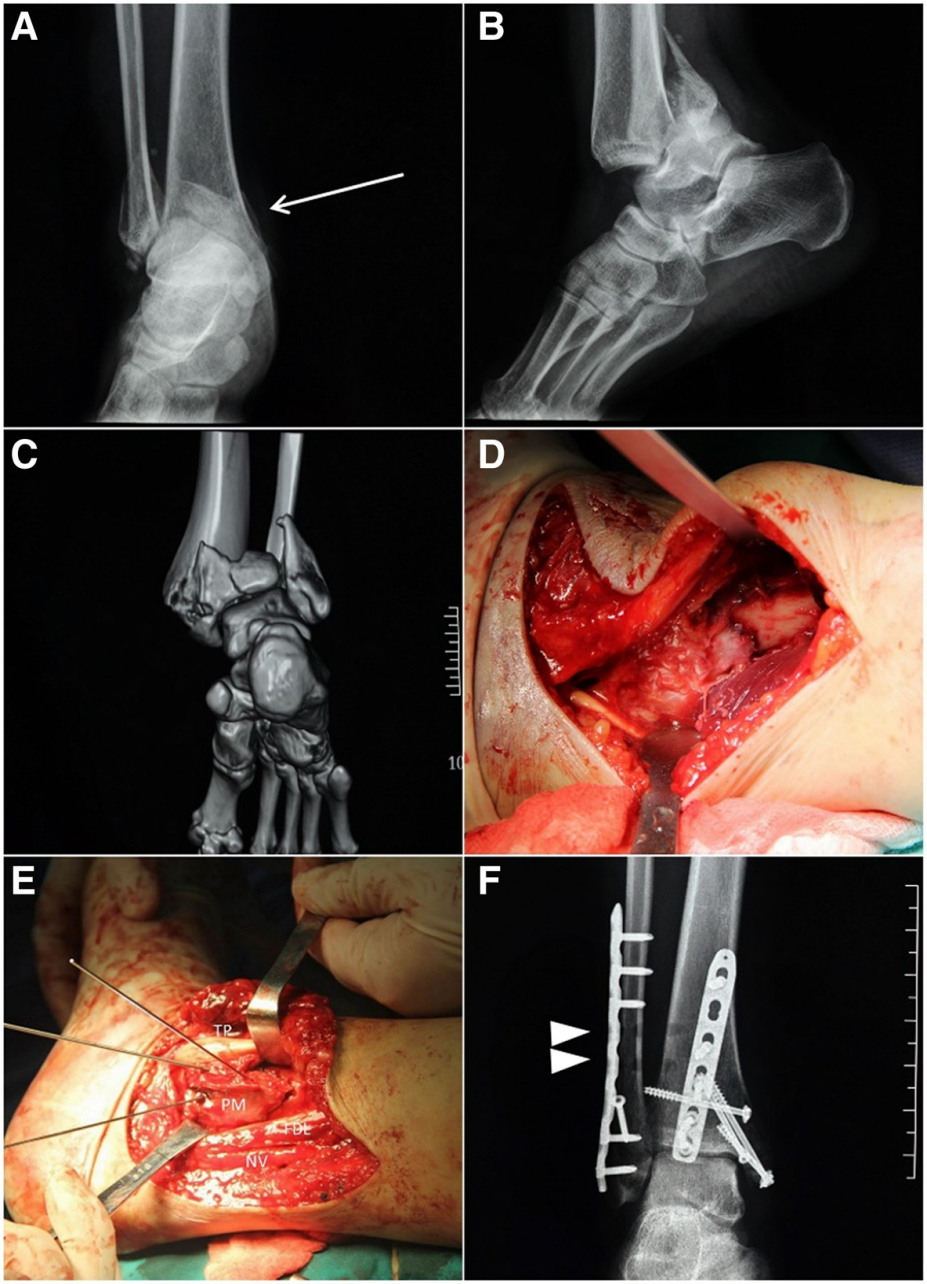
Illustration figures of case 1, with talar subluxation, syndesmotic disruption, posteromedial fracture involving posterior colliculus and complete medial malleolar fracture. **a**-**c** Preoperative images of posterior pilon variant. The *Arrow* shows the “double contour” sign. **d** Exposure of the PL fragment through the plane between NV bundle and FHL. The hohman retractor was placed on the tibia, gently blocking the NV from injury. **e** Temporary fixation of the PM fragment with TP tendon subluxated medially and FDL retracted laterally. **f** As the stress test was found positive, absorbable syndesmotic screws were placed. (*Arrow Head*) Noticing the buttress plate for PL fragment was placed obliquely, which was medial proximally and lateral distally

**Fig. 3:**
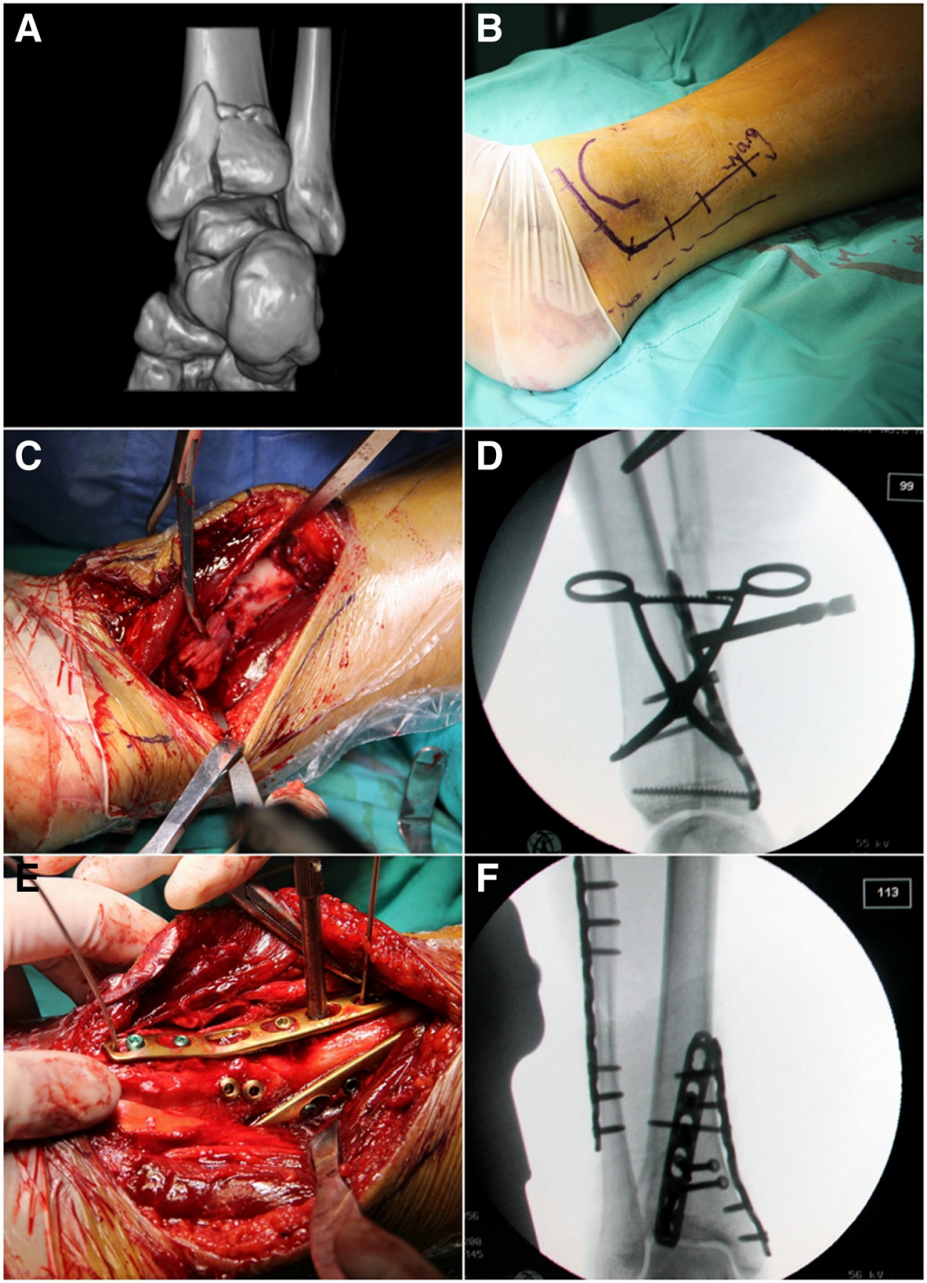
Illustration figures of case 7. **a** The fracture line of PM fragment did not extend into posterior colliculus but the size of PM fragment was relatively large. **b** The modified PM incision which was composed of vertical and transverse branch. The Achilles tendon (*dotted line*) and medial malleolus were also marked. **c**, **d** Reduction of the PL fragment: using a large periarticular clamp. After contoured buttress plate was placed, the intraoperative fluoroscopic image of lateral view was taken. **e** A medially based plate and two posterior-to-anterior lag screws were used to stabilize the PM fragment. **f** Final intraoperative fluoroscopic image of the patient. The PL buttress plate was place obliquely

**Fig. 4:**
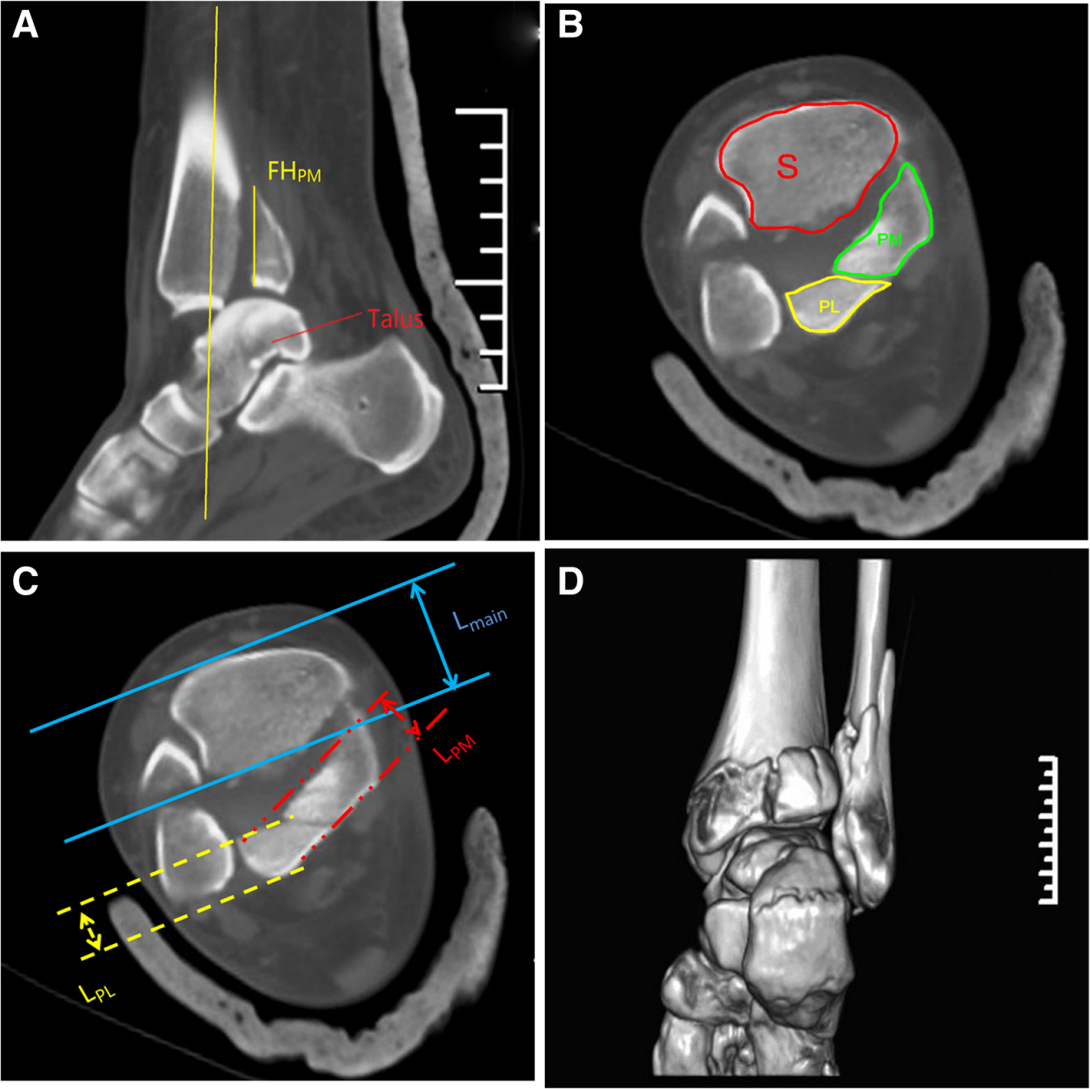
Radiographic measurements. **a** The largest distance from the apex of the posteromedial or posterolateral fragment to the the articular surface on consecutive sagittal reconstruction views is defined as the fragment height (FH). **b** The posteromedial and posterolateral fragment area ratio (FAR) was determined by calculating the percent of area (PM/PL)/area (PM+PL+ S). **c** The posteromedial and posterolateral fragment length ratio (FLR) was determined by calculating the percent of L_PM/PL_/ L_Main_+L_PM/PL_. **d** The CT reconstruction of the same Case

Institutional review board approval of Shanghai Sixth People’s Hospital was obtained before the initiation of this study. From January 2010 to January 2012, 16 posterior pilon variant cases treated via modified posteromedial approach at our level I trauma center were included in the study. The diagnosis was confirmed based on the “double contour” sign on the AP views and “double joint line” on the lateral view [2, 12, 13] (Figs. 1a-b and 2a-b). CT scan was obtained to determine the comminution and impaction of the posterior tibial plafond (Figs. 1c-d, 2c and 3a). Morphologic characteristics of the posterior tibial plafond fragments, including the fragment height(FH), fragment length ratio(FLR) and fragment area ratio(FAR) [17], were measured according to the Haraguchi’s study via Picture Archiving and Communication System (PACS) [4, 18] (Fig. 4). All fractures underwent reduction and fixation via modified posteromedial approach. Additional lateral incision was made only for fibular fixation (Table 1). Reduction of the fracture and functional outcomes were presented in Table 2.

**Table 1:** Demographics & injury characteristics

N.	Sex/Age	Fib. Fx.	PM Fx. Ex.	MM Fx.	Syn. Dis.	Injury mechanism	AO/OTA 44-	Lange Hansen	Talar Sublux.	Plaster/Traction	Waiting days
1	F/45	B	Y	Y	Y	MVA	B3.2	SER IV	Y	T	9
2	F/38	B	Y	Y	N	FFH	B3.2	SER IV	Y	T	8
3	F/51	B	Y	Y	N	PFF	B3.2	SER IV	N	/	8
4	M/48	B	Y	Y	N	FFH	B3.2	SER IV	Y	P	8
5	F/53	B	Y	N	N	PFF	B3.2	SER IV	Y	P	10
6	F/45	B	Y	Y	N	MVA	B3.2	SER IV	Y	P	9
7	M/68	C	N	N	Y	MVA	C2.3	PER IV	N	/	8
8	F/37	B	Y	Y	N	PFF	B3.2	SER IV	Y	T	9
9	F/55	B	N	Y	N	FFH	B3.2	SER IV	N	/	8
10	F/45	B	Y	Y	N	MVA	B3.2	SER IV	Y	T	7
11	F/68	C	Y	Y	N	MVA	C2.3	PER IV	N	/	9
12	M/41	C	N	N	N	FFH	C2.3	PER IV	N	/	10
13	F/38	B	Y	Y	N	PFF	B3.2	SER IV	Y	P	9
14	F/44	B	Y	Y	N	MVA	B3.2	SER IV	Y	P	8
15	F/63	C	N	N	Y	MVA	C2.3	PER IV	Y	T	10
16	M/48	B	Y	Y	N	FFH	B3.2	SER IV	N	T	9

*Fib. Fx.* fibular fracture type using Weber classification, *PM Fx. Ex.* medially based wedge posteromedial fragment extending into posterior colliculus of medial malleolus, *MM Fx.* complete medial malleolus fracture involving both anterior and posterior colliculus, *Syn. Dis.* syndesmotic disruption; injury mechanism, *MVA* motor vehicle accident, *FFH* fall from a height, *PFF* plantar flexion when fall, *Talar Sublux.* talar subluxation

**Table 2:** Clinical & radiological outcomes

NO	Operation time (mins)	Fracture healing (wks)	VAS			AOFAS	OA score	Follow up (mos)
			Rest	Motion	WB walking			
1	105	13	0	1	2	85	1	28
2	90	14	2	3	4	77	2	36
3	95	12	0	0	1	95	0	28
4	105	12	1	2	2	78	2	40
5	95	12	0	0	0	90	0	34
6	100	14	0	0	0	94	0	24
7	105	13	0	0	1	91	0	36
8	100	12	0	0	1	92	0	30
9	115	14	1	2	2	78	2	28
10	110	17	0	0	1	93	0	34
11	105	14	0	1	2	79	1	30
12	110	12	0	1	2	NA	NA	4
13	100	12	0	0	0	NA	NA	6
14	100	13	0	1	1	79	1	24
15	100	14	0	1	0	82	1	24
16	95	12	0	1	2	85	0	26
Mean	101.8	13.1	0.25	0.81	1.31	85.6	0.71	30.1

Statistical analyses regarding the fragment measurement were performed using SPSS 19.0 software (SPSS Inc., Chicago, IL, USA). The descriptive statistics were employed.

## Surgical technique

### Preparation and exposure

The patient was positioned supine with a tourniquet on the thigh on a radiolucent table. The operative limb was placed in a letter D position, and the ankle was externally rotated with a bump placed underneath. This position facilitate later steps to access both PM and PL fragments. The skin incision started longitudinally along the medial border of the Achilles tendon, and then curved at the plane distal to medial malleolus, following toward the talonavicular joint. The length of the incision was dependent on the metaphyseal extension of the fracture (Fig. 3b). The flexor retinaculum was incised lateral to the flexor hallucis longus tendon (FHL).

### Approach to posterolateral fragment

PL fragment was approached first through the plane between FHL and neurovascular (NV) bundle. Care must be taken to protect the NV bundle, using a hohman retractor gently block it medially together with Tibialis Posterior tendon (TP) and flexor digitorum longus tendon (FDL) [19] (Fig. 2d). Dissection was continued proximally through this plane. In cases when comminution or impaction occurs, the fragments were opened like a book as its lateral hinge remained [11]. The PL fragment can be reduced using a ball spike or a large periarticular clamp placing around to the anterior tibial surface (Fig. 3c). Provisional 2.0-mm K-wires were used to stabilize the fragments before definitive 3.5 mm buttress plate fixation. The buttress plate was placed in an oblique fashion (Figs. 2f and 3f).

### Approach to posteromedial fragment

The second plane between FDL and TP could expose the PM tibial plafond. After the tendon sheaths were incised in line with its underlying tendon, the FDL was retracted laterally to protect the NV bundle, while the TP tendon was mobilized and subluxated medially over the medial malleolus. Continuing sharp dissection over the floor of the tendon sheath will expose the PM fragment. After reduction, either multiple 3.5 mm lag screws or low-profile buttress plate could be used as final fixation according to the fragment size (Figs. 2e and 3e).

### Further exposure

If there were a separate fragment in anterior colliculus or a complete medial malleolus (MM) fracture, the TP was put back to its original position making the whole MM under direct visualization, which constituted the third plane. Lag screw fixation would be applied if both the anterior and posterior colliculus were involved. Intraoperative radiographs are evaluated in each fragment fixation to confirm the correct reduction. Fibular reduction and fixation were approached last through a lateral incision with the operative limb turned into neutral position. Syndesmotic screws would be placed if the stress test were positive intraoperatively (Fig. 2f). The tendon sheath and flexor retinaculum were repaired before wound closure.

### Postoperative management

Postoperatively, posterior splint was applied and all patients were kept non-weight bearing for at least 4 weeks. The splint was removed at 2–3 weeks, at the same time, active motion exercises initiated. Permission to full weight bearing depended on radiographic and clinical signs of healing, usually 12 weeks postoperatively.

## Results

There were 12 females and four males, and the mean age at the time of injury was 49.2 (range, 37–68) years. Fourteen out of 16 cases constituted clinical outcomes because the remaining two were lost follow up. The mean follow-up time was 30.1 months. Subluxation of the talus occurred in 10 of 16 cases, and preliminary closed reduction with plaster stabilization was taken in five cases. The other six took calcaneal traction after reduction (see Additional file 1: Figure S5 and Additional file 2: Figure S2). Modified posteromedial approach combined with separate lateral approach (for fibular fixation) was used in all patients. Additional PM buttress plate was used in five cases and syndesmotic screw fixation was applied in four cases.

### Morphological characteristics

Axial and coronal images revealed that fracture line extending from PM to medial malleolar (MM) fragment existed in 12 out of 16 cases. All cases had associated lateral malleolar fracture and 12 of them had complete MM fracture involving both anterior and posterior colliculus [12]. The results of morphological characteristics such as FH, FLR and FAR were listed in Table 3 (Fig. 4).

**Table 3:** Morphologic characteristics of the posteromedial and posterolateral fragment in posterior pilon variant

	PM fragment	PL fragment	PM+PL
FLR(%)			
Mean ± SD	25.3 ± 5.1	31.5 ± 4.8	NA
Range	17.5–35.5	23.0 ± 39.3	NA
FAR(%)			
Mean ± SD	16.1 ± 3.8	15.5 ± 3.1	31.6 ± 3.1
Range	10.3–20.5	10.3–19.6	26.9–36.9
FH(mm)			
Mean ± SD	23.5 ± 10.0	23.8 ± 6.4	NA
Range	8.5–42.5	12.5–32.5	NA

*FLR* fragment length ratio, *FAR* fragment area ratio, *FH* fragment height

### Clinical outcomes

No delayed or nonunion was found. Accurate reduction was achieved in all patients (articular step-off less than 2 mm) based upon the comparison between radiographs taken immediate and sixth-month postoperatively. No wound complication or hardware irritation was found. The American Orthopaedic Foot & Ankle Society (AOFAS) ankle/hindfoot questionnaires were completed at 24-month follow-up (Table 2). No tendon contraction was found.

## Discussion

Posterior pilon variant fracture is a recently defined challenging fracture, which cannot exactly fall into either of the categories: pilon fracture or malleolar fracture. Its uniqueness in injury mechanism and fracture pattern could distinguish itself from the above two [1, 2, 5, 12–14].

Unlike classic pilon fractures, about 8 days’ waiting period for soft tissue resolution in Chen’s and in this study [14] both indicate that posterior pilon variant is not from high energy trauma that requires staged management in pilon fracture [20, 21]. Besides, the coronal fracture lines found in posterior pilon variant was different from sagittal fracture lines in high energy pilon fracture described by Topliss et al. [22]. On the contrary, the fracture lines were consistent with the fracture map of posterior malleolus [3, 4]. What’s more, unlike malleolar fracture caused by low energy torsional force, the independent PM fracture in posterior pilon variant not only extends proximally but also often involves posterior colliculus of medial malleolus [3, 4, 12–15], which is 12 out of 16 in this study. In pathoanatomy studies focusing on posterior malleolar fractures [3, 4], those posterolateral (PL) fractures with transverse medial extension were classified as Haraguchi Type II, which has a 29.8 % involvement of tibial plafond area [4]. In this study, the total fracture area involved in posterior pilon variant is larger than malleolar fracture but close to pilon fracture, which is 31.7, 13.7 and 30.3 % respectively [18]. Additionally, talar subluxation was found common (10/16) in posterior pilon variant as well [4, 14].

The description of posterior pilon variant can be summarized as followed: the injury level which lies between low energy torsion and high energy compression causes proximally displaced (and impacted) posterior tibial plafond fracture. Two main fragments (PM and PL) exist. The fracture line of PM fragment usually extends into posterior colliculus of medial malleolus. In most cases, lateral malleolus is also fractured. The radiological sign of “double contour” and “double joint line” sign on AP and lateral view, both indicating the presence of posterior pilon variant [1–3, 10, 11] (Figs. 1a-b and 2a-b).

Posterolateral approach in prone position which was initially designed for posterior malleolus fracture, is the most accepted surgical approach to posterior pilon variant at present. Additional limited posteromedial incision is made only when PM fragments could not be accessed through the posterolateral incision [6, 11, 12, 14, 16]. Complications such as sural nueritis and regional pain were reported using posterolateral approach [12]. Moreover, recent cadaveric study [23] showed the potentially high risk of injuring the perforating branch of peroneal artery using posterolateral incision: the safe distance could be as limited as 41 mm. Based on our clinical experience, we found it hard to manipulate both PL and PM through the single posterolateral incision, as either the attachment to deltoid ligament or the entrapment of soft tissue may prevent PM fragment from anatomical reduction [13, 15, 24]. In comparison to the reduction of PL fragment, which can be achieved through ligamentotaxis, direct visualization is always required reduce PM fracture.

To lower the risk of various complications and facilitate exposure, modified posteromedial approach was applied in this study [7, 25]. The approach was characterized by direct handling PM and PL fragments of posterior tibial plafond through three different anatomic planes in supine position. The transverse branch of the incision is almost in line with the medial incision for talus neck fracture [23], while the vertical branch is medial to the Achilles tendon and extends proximally based on metaphyseal involvement. Though never reported in posterior pilon variant fracture, it may take advantage in the following three aspects over posterolateral approach. First, the approach has a lower risk of injuring perforator branch of peroneal artery, which was 61 mm to tibial plafond on average [26]. The placement of buttress plate was the key step. When using modified posteromedial approach, the plane developed between FHL and NV bundle allowed buttressing the PL fragment *obliquely*, which meant placing the plate proximally medial and distally lateral (Figs. 2f and 3f). Second, the anatomic safety is further guaranteed by incision design. Modified PM incision curves above the three main branch of posterior tibial artery, the angiosomes of medial calcaneal and plantar are safe with meticulous protection of full thickness fasciocutaneous flap [27]. Besides, as the whole posterior tibial plafond could be accessed through the same PM incision, lateral approach to the lateral malleolus is preferred, leaving a larger skin bridge. Third, supine position had less anesthesia related complications and better alignment measurement [28]. As the position facilitates intraoperative fluoroscopic evaluation of lower limb axis as well as joint surface, it raised efficiency as well.

## Conclusion

The results from our study regarding patients’ age, fracture reduction, bone healing, and functional outcomes are consistent with related studies on posterior pilon variant fracture or posterior malleolar fracture [5, 12, 14, 15]. In conclusion, we considered it is a safe and alternative approach to treat posterior pilon variant fracture via modified posteromedial approach. It provides adequate visualization, direct reduction, stable fixation and good short-term outcomes.

## Abbreviations

AOFAS, American Orthopaedic Foot & Ankle Society; FAR, fragment area ratio; FDL, flexor digitorum longus; FH, fragment height; FHL, flexor hallucis longus tendon; FLR, fragment length ratio; MM, medial malleolus; NV, neurovascular; PACS, picture archiving and communication system; PL, posterolateral; PM, posteromedial; TP, tibialis posterior

## Additional files

Additional file 1: Figure S5. Calcaneal Traction after initial evaluation. (TIF 4243 kb) Additional file 2: Figure S6. Cast stabilization after initial reduction. (TIF 5880 kb)
