# Supplementary figures and images for: Modified posteromedial approach for treatment of posterior pilon variant fracture
Source: BMC Musculoskelet Disord. 2016 Aug 5;17:328. doi: 10.1186/s12891-016-1182-9 (PMC4974710; doi:10.1186/s12891-016-1182-9)

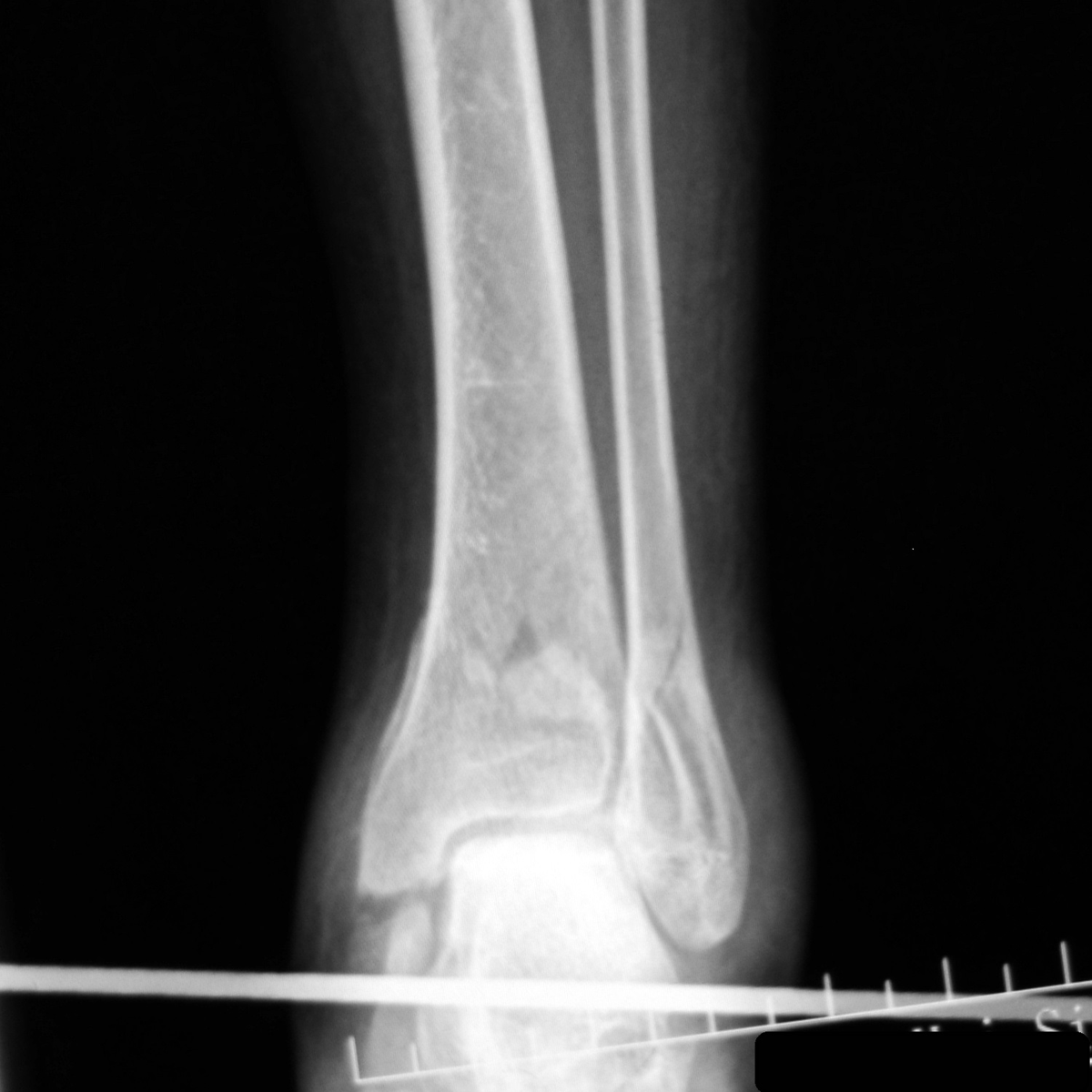

Supplement: Additional file 1: Figure S5. — Calcaneal Traction after initial evaluation. (TIF 4243 kb) [file 12891_2016_1182_MOESM1_ESM.tif]

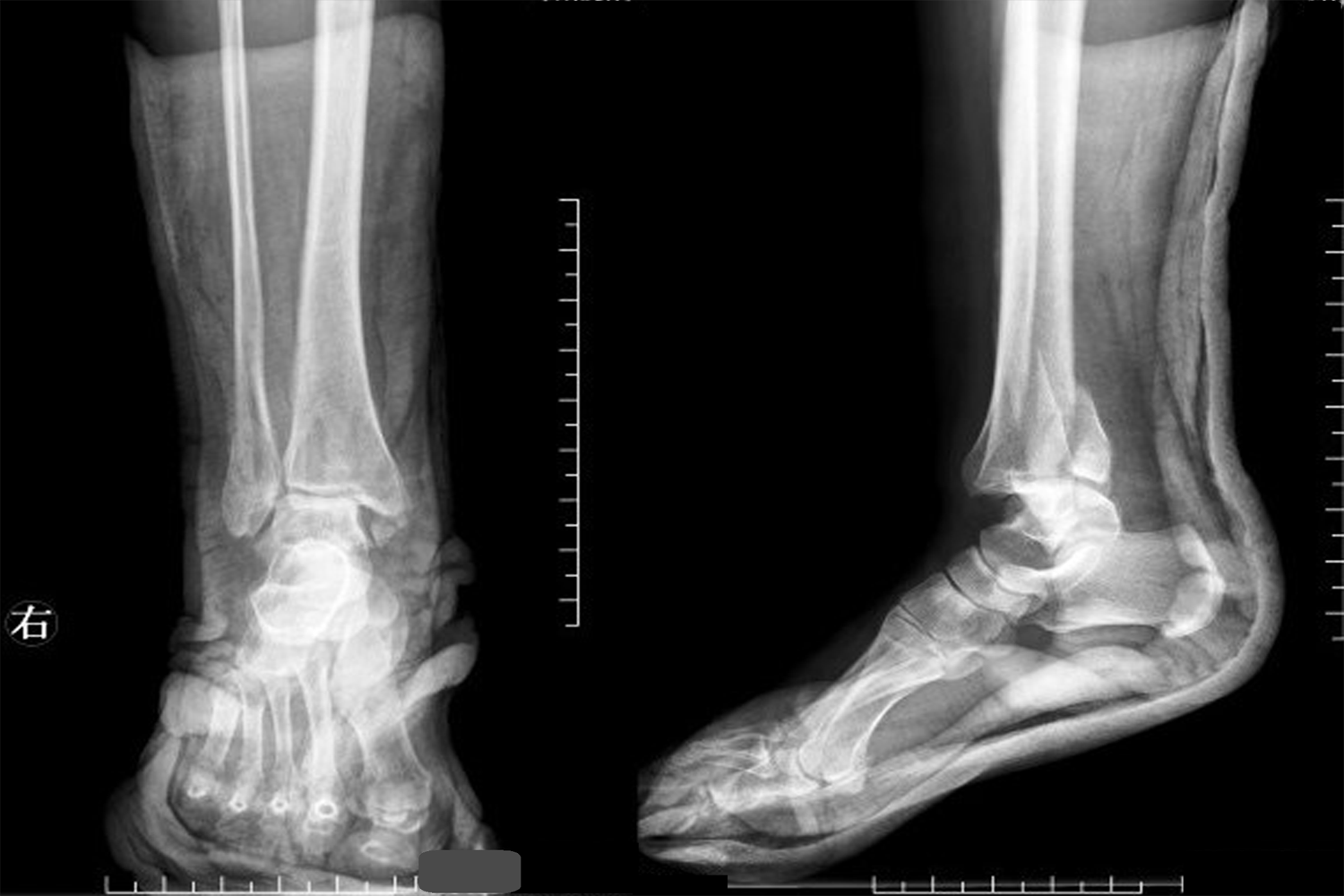

Supplement: Additional file 2: Figure S6. — Cast stabilization after initial reduction. (TIF 5880 kb) [file 12891_2016_1182_MOESM2_ESM.tif]
